# Changes in functional brain network topology after successful and unsuccessful corpus callosotomy for Lennox-Gastaut Syndrome

**DOI:** 10.1038/s41598-018-21764-5

**Published:** 2018-02-21

**Authors:** Jun-Ge Liang, Nam-Young Kim, Ara Ko, Heung Dong Kim, Dongpyo Lee

**Affiliations:** 1 RFIC Center, Kwangwoon University, Seoul, Republic of Korea; 2 Epilepsy Research Institute, Yonsei University College of Medicine, Seoul, Republic of Korea; 3 Department of Pediatrics, Pediatric Epilepsy Clinic, Severance Children’s Hospital, Yonsei University College of Medicine, Seoul, Republic of Korea

## Abstract

Corpus callosotomy (CC) is an effective palliative surgical treatment for patients with Lennox-Gastaut Syndrome (LGS). However, research on the long-term functional effects of CC is sparse. We aimed to investigate these effects and their associated clinical conditions over the two years after CC. Long-term clinical EEG recordings of 30 patients with LGS who had good and bad seizure outcome after CC were collected and retrospectively studied. It was found that CC caused brain network ‘hubs’ to shift from paramedian to lateral regions in the good-recovery group, which reorganized the brain network into a more homogeneous state. We also found increased local clustering coefficients in patients with bad outcomes and decreases, implying enhanced network integration, in patients with good outcomes. The small worldness of brain networks in patients with good outcomes increased in the two years after CC, whereas it decreased in patients with bad outcomes. The covariation of small-worldness with the rate of reduction in seizure frequency suggests that this can be used as an indicator of CC outcome. Local and global network changes during the long-term state might be associated with the postoperative recovery process and could serve as indicators for CC outcome and long-term LGS recovery.

## Introduction

Lennox-Gastaut Syndrome (LGS) is an epileptic disorder that arises in childhood and is typically characterized by intractable, multiple, generalized seizure types and electroencephalograms showing slow spike-and-wave patterns, generalized polyspikes, and a slow background^1,2^. LGS accounts for approximately 1–10% of all childhood epilepsies, and it is a rare but catastrophic childhood epileptic encephalopathy due to its deleterious effects on intellectual and psychosocial function^3,4^. Brain dysfunctions such as LGS are increasingly seen as consequences of a complex interplay of dynamic neural systems overlaid on functional networks. Disruptions in these networks may be associated with cognitive, behavioural, and neurodevelopmental impairment related to epilepsy^5,6^. Some studies have found that intracranial recording during ictal periods revealed a network shift towards a more regular topology than that observed in partial seizures^7^, and that an increased clustering coefficient and path length can mark seizure onset. Thus, functional neural network analysis is a promising technique for more accurate identification of the target areas for epilepsy^8–10^.

As the most typical structure in network organization, small-world topology can balance the properties of integration and separation to achieve efficient internal communication^11–13^. This topic has been discussed in many different publications. Warren and colleagues showed that a large increase in the clustering coefficient and stable path lengths indicate departure from a random to small-world topology in brain networks of patients with LGS^14^. Other studies of temporal lobe epilepsy^15^, Alzheimer’s disease^16^, and attention-deficit hyperactivity disorder^17^ have also shown a reduction in small-worldness in the brain network. Epileptic networks can also be characterized by a small-world topology. According to an epilepsy simulation study conducted by Netoff and colleagues, the ‘seizing’ behaviour of the CA1 region corresponds with small-world topology^18^. In another study of temporal lobe epilepsy, brain networks in patients were characterized by a short path between anatomical regions and a high degree of clustering, suggestive of a small-world network^19^. In view of its demonstrated value in describing brain status, small-worldness could serve as a quantifiable indicator to evaluate brain states, combining both local and global network characteristics.

Because LGS is generally intractable to medical treatment and sometimes not amenable to focal resection, corpus callosotomy (CC) is frequently performed as a palliative treatment^20–22^. It is thought that CC alleviates seizures because the corpus callosum is a crucial connection for the spread of epileptic activity^23^. Over the years, CC has been confirmed to be remarkably effective for a variety of intractable seizures and epilepsy syndromes, particularly tonic, atonic, and tonic-clonic seizures. Many retrospective studies have been conducted to study CC treatment of LGS, showing that patients were able to achieve over 50% reduction in seizure frequency^22^. Moreover, studies examining the long-term follow-up statistics of seizure outcomes for more than 3 years after CC show favourable outcomes. The seizure-free rate for drop attacks and other types of seizures was around 90% after complete callosotomy, whereas a lower seizure-free rate of 54% was obtained for partial resection^24^.

Most of these studies focused on the effectiveness and rate of successful rehabilitation after CC. However, the influence of CC on cerebral activity, including brain network effects and their relations to LGS recovery, has not been extensively studied. We previously published a baseline study of the short-term functional network effects of CC in patients with Lennox-Gastaut syndrome^25^. In that study, we gained insight into the connectivity state change right after CC and found a less synchronized brain network with more of a small-world topology. However, as a pilot study, there were some limitations, including the absence of an in-depth discussion of the CC mechanism and the lack of a quantitative analysis of important graph structure indicators such as the clustering coefficient and small-worldness. The study focused on the immediate effects after CC and did not provide evidence for long-term network changes. The lack of a control group in the previous study also weakened our claims. We addressed these shortcomings by comparing the results between groups with good and bad seizure outcomes for up to two years following the CC.

In the current study, we aimed to investigate the functional network effects of CC in patients with LGS and the continuity of these effects in a long-term follow-up. We analysed the representative brain network first in the preoperative state and later at 3-month, 1-year, and 2-year post-operation. Several network measures, including global, local measures and small-world organization, were combined to evaluate the effects of CC on functional networks and their association with the long-term recovery process.

## Results

### Enhanced postoperative homogeneity of network topology

**Figure 1:**
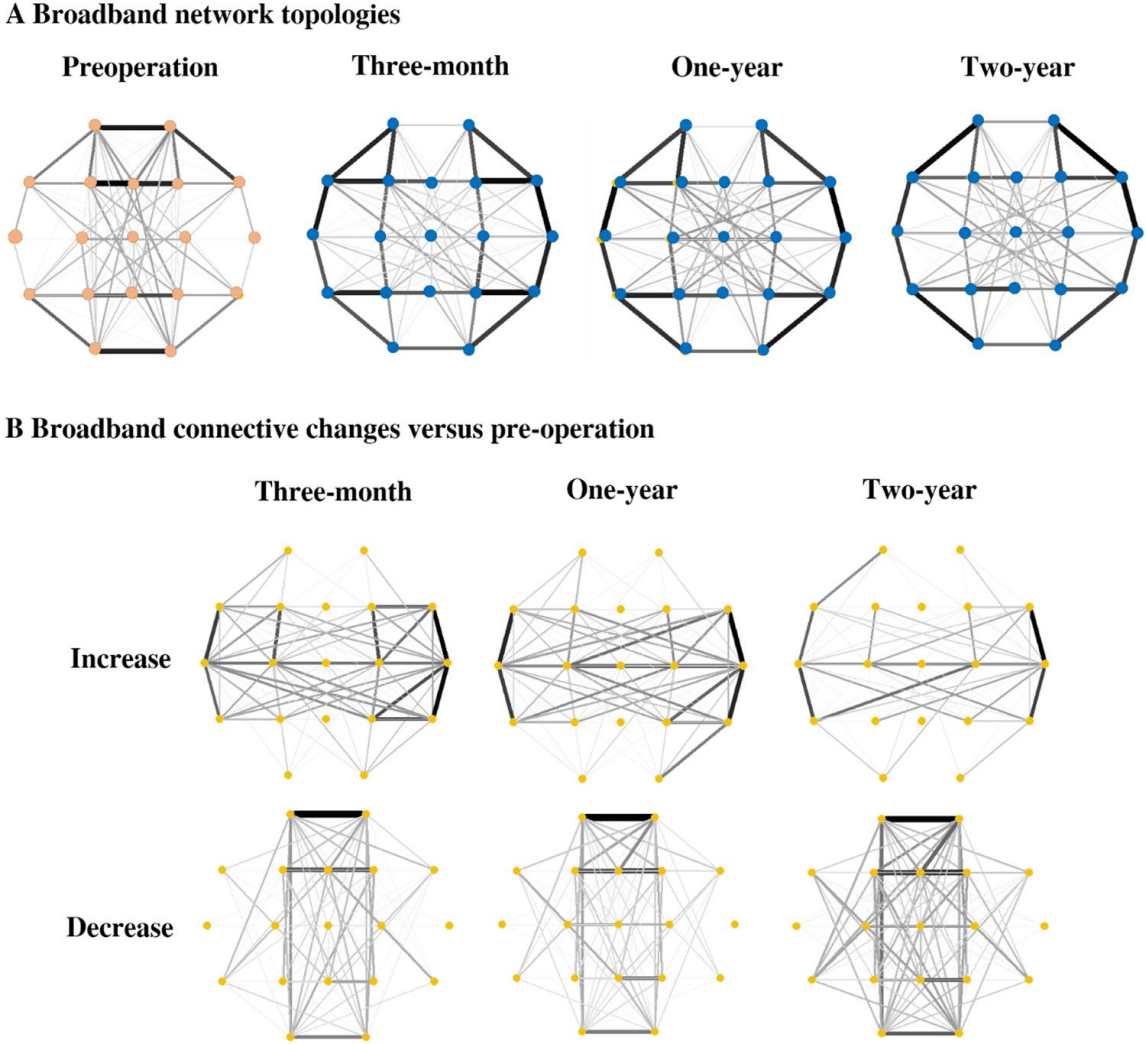
Broadband network topologies and the changes from pre-operation to three-month, one-year, and two-year postoperative states. (**A**) In the preoperative state, most connections located around the paramedian regions notably weakened after CC; this change generally remained consistent at three-months, one- and two-years post-operation. (**B**) After CC, the interhemispheric connections increased and the paramedian connections decreased; these changes persisted in the long-term recordings. The decreased connectivity at two-years was greater than the other changes.

Figure 1A shows the changes in patterns of broadband network topologies between pre- and post-operation states. In the preoperative state, most connections were located around the paramedian brain regions. These connections notably weakened after CC, whereas the connections in lateral regions showed enhancement and the network topology changed into a more homogeneous state (Supplementary Figure S1). This replicates our previous findings^25^, and the changes generally remained consistent at one- and two-years post-operation.

The increased and decreased connectivities at three-months, one-year, and two-year post-operation versus pre-surgical network connectivity are depicted in Fig. 1B. After CC, the interhemispheric connections increased in strength and density, and this general pattern remained at the two-year postoperative follow-up. Additionally, the connections in the paramedian regions decreased, and this change also persisted in the long-term recordings. Nevertheless, the decrease in functional connectivity at the two-year follow-up was greater than the other changes.

### Postsurgical hubs moved from paramedian to lateral regions

**Figure 2:**
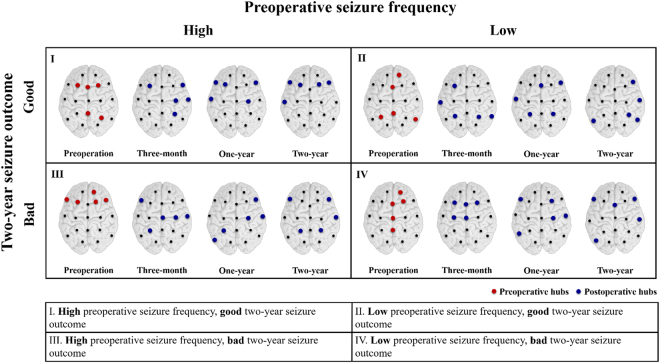
Hubs derived from betweenness centrality (BC) intensity for the four groups (See Supplementary Figure S3 for details). In the good outcome groups, I and II, the high BC values and the hubs were mainly distributed around the paramedian region before CC, and shifted to more lateral regions after CC. In group III, the High-Bad group, the preoperative hubs mostly focused on the frontal regions. In group IV, the Low-Bad group, although the preoperative hubs were concentrated in the midline regions, their locations were not greatly affected by CC; they were still located near the midline regions in the three-month state.

Hubs are the crucial nodes that facilitate the spread of EEG activity in the network. In Fig. 2, the nodes with the five largest BC values are selected as the hubs and plotted in red and blue colours. In the good outcome groups, regardless of whether the preoperative seizure frequency was high or low, the high BC values (the hubs) were mainly distributed around the paramedian region before CC, and shifted to more lateral regions after CC. However, in the High-Bad group (III), the preoperative hubs were initially distributed around the frontal regions, and CC shifted them to posterior regions. In the Low-Bad group (IV), the locations of hubs did not change clearly over the three months after CC. In summary, CC caused consistent and significant hub movements in the good outcome groups, but the changes were not as consistent in the bad outcome groups.

### Postsurgical changes in small-world organization

**Figure 3:**
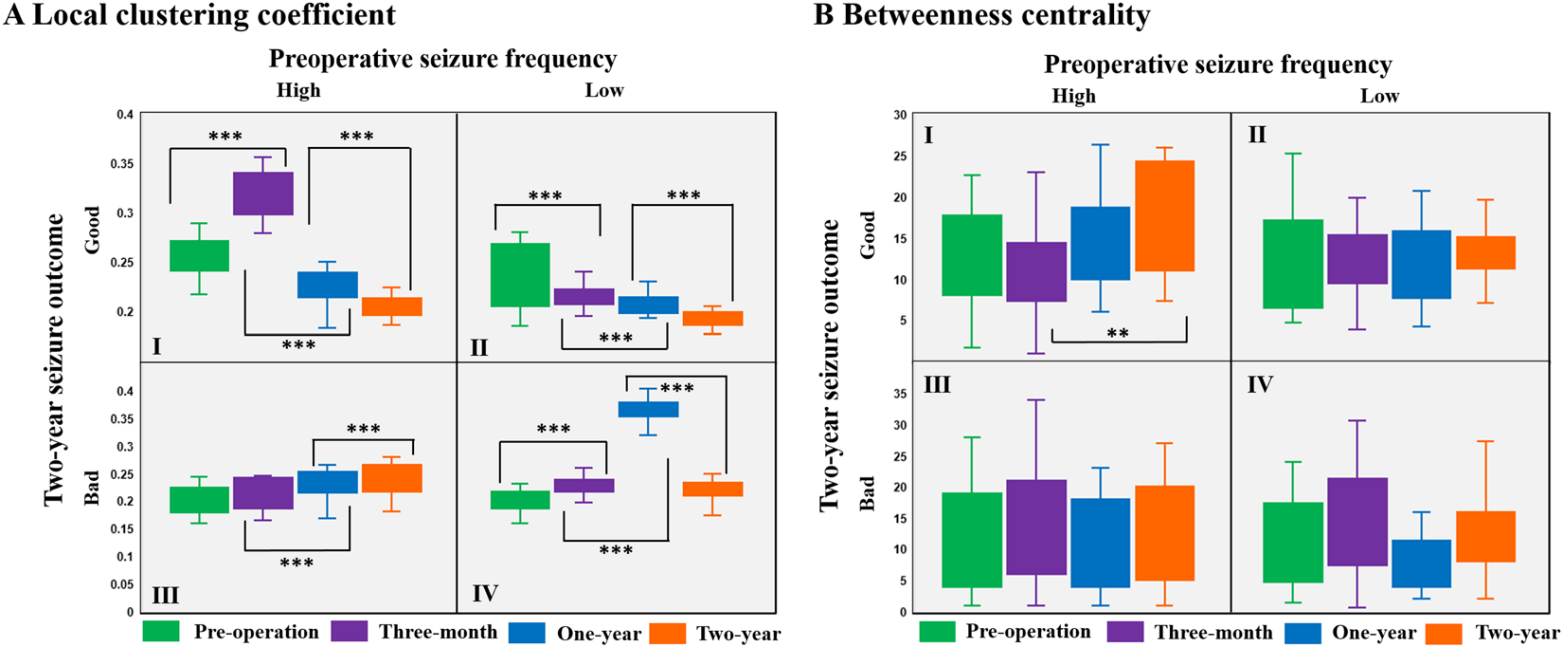
Comparisons of local network parameters including (**A**) local clustering coefficient (LCC) and (**B**) betweenness centrality (BC). Patients in group I showed increased LCC (*p* < 0.001) three-months after surgery and decreased LCC (*p* < 0.001) at one-year and two-year post-operation (repeated measures ANOVA, *F*_3,15_ = 3.876, *p* = 0.031, Bonferroni post-hoc tests). For group II, LCC decreased (*p* ≤ 0.001) at the two-year follow-up compared to the pre-operation value (repeated measures ANOVA, *F*_3,30_ = 3.022, *p* = 0.045, Bonferroni post-hoc tests). Both groups III and IV showed an increased LCC (*p* < 0.001) after CC at one-year follow-up; group III increased further at the two-year state (repeated measures ANOVA, *F*_3,15_ = 4.101, *p* = 0.026, Bonferroni post hoc tests) whereas group IV decreased (repeated measures ANOVA, *F*_3,18_ = 4.495, *p* = 0.016, Bonferroni post hoc tests). The LCC in a fully-connected network equals 1. BC in Group I slightly decreased three-months after CC and then showed a significant increase (*p* < 0.05) after two years of recovery, whereas no clear changes were seen in the other groups (repeated measures ANOVA, *F*_3,15_ = 4.006, *p* = 0.028, Bonferroni post hoc tests).

**Figure 4:**
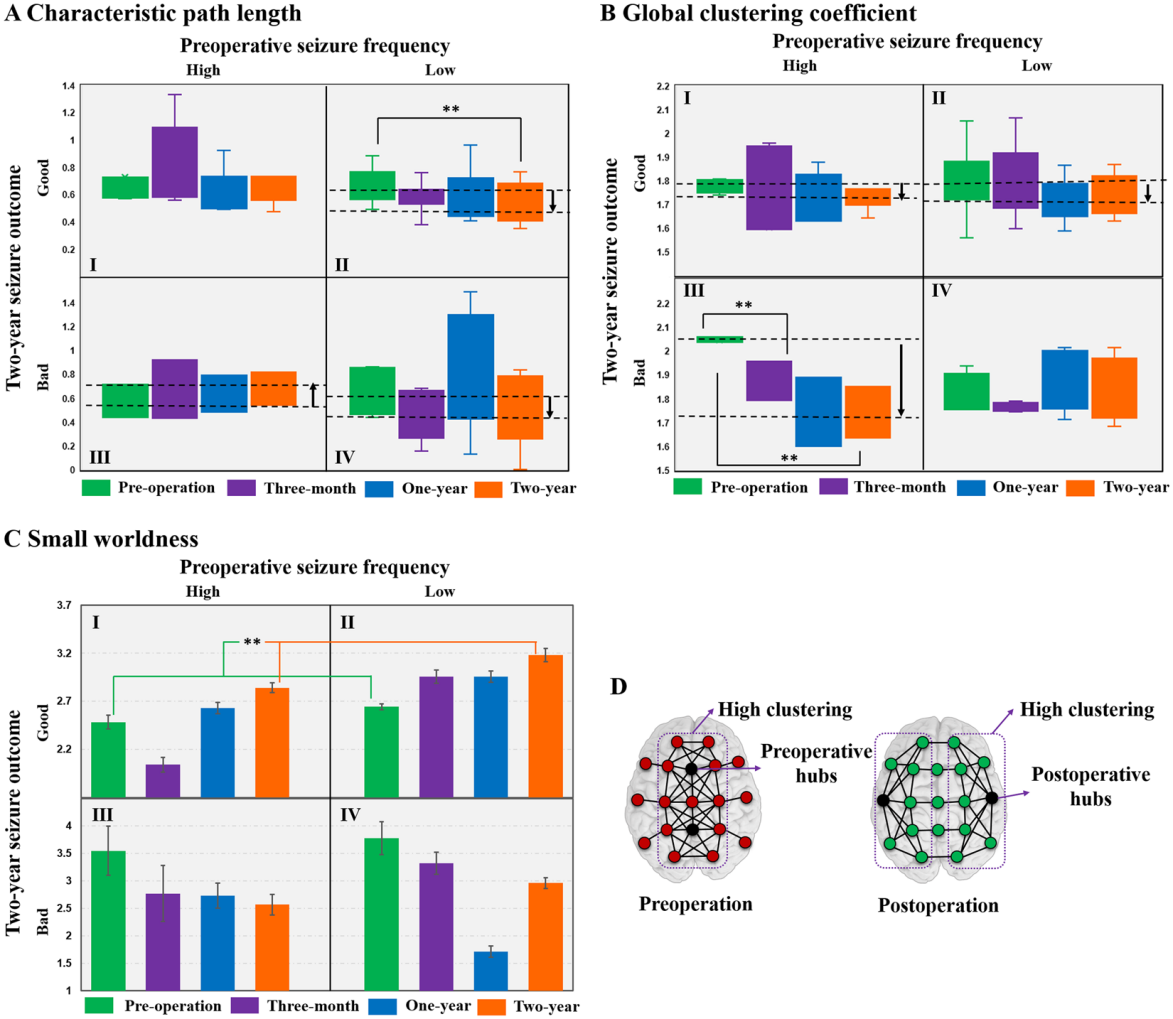
Comparisons of global parameters including (**A**) characteristic path length (CPL) and (**B**) global clustering coefficient (GCC). In group II the CPL showed a decrease at the two-year follow-up (*p* < 0.05) when compared to the pre-operation value (repeated measures ANOVA, *F*_3,30_ = 3.209, *p* = 0.037, Bonferroni post hoc tests), while group III showed an increasing trend and group IV showed a decreasing trend. A notable change was seen in the global clustering coefficient (GCC) of group III, in that the GCC continuously decreased (*p* < 0.05) for two years after CC (repeated measures ANOVA, *F*_3,15_ = 4.382, *p* = 0.021, Bonferroni post hoc tests). GCC also showed a decreasing trend in groups I and II. (**C**) Small-worldness calculated as the ratio of the global clustering coefficient to characteristic path length (error bars: standard errors). None of the groups showed statistically significant differences. However, the small-worldness of patients with good outcomes (groups I and II combined, repeated measures ANOVA, *F*_3,48_ = 3.621, *p* = 0.038, Bonferroni post hoc tests) increased (*p* = 0.03) two years after CC whereas it decreased in patients with bad outcomes. The small-worldness of a fully-connected network equals 1. (**D**) Simplified illustration of functional network pattern changes from pre- to post-operation.

We calculated two local network measures, local clustering coefficients (LCCs) and betweenness centrality (BC), and show box diagrams for them in Fig. 3A, and repeated measures ANOVA analyses in Fig. 3B. As described in the Methods section, the patients were classified into four groups, I, II, III, and IV, based on the preoperative daily seizure frequency and its reduction rate after a two-year recovery. Patients in group I (High-Good) showed a significantly increased LCC three months after surgery and an evident decrease at one-year and two-year post-operation. For group II (Low-Good), LCC significantly decreased at the two-year follow-up compared to the pre-operation value. Both groups III (High-Bad) and IV (Low-Bad) showed a significantly increased LCC after CC at the one-year follow-up: the value for group III continuously increased over two years whereas the value for group IV decreased. Thus, the good-recovery groups (I and II) showed significantly decreased LCC after two years, whereas the bad-recovery groups (III and IV) showed increased LCC values. BC in Group I slightly decreased three months after CC and then showed significant increase after two years of recovery, whereas no clear changes were shown in the other groups. As for the global parameters, characteristic path length (CPL) in group II showed a significant decrease at the two-year follow-up when compared to its pre-operation value, while group III showed an increasing trend and group IV showed a decreasing trend (Fig. 4A). A notable change was seen in the global clustering coefficient (GCC) of group III, in that the GCC continuously decreased for two years after CC (Fig. 4B). GCC also showed a decreasing trend in groups I and II, but without statistical significances.

The variation of the small-worldness (calculated as the ratio of GCC to CPL) is summarized in Fig. 4C. In group I, the value decreased three months after CC, then increased, and the value at the two-year follow-up was higher than pre-operation. Small-worldness in group II showed a gradual increase after CC, and the values in each state were relatively higher than group I. Groups III and IV showed relatively lower small-worldness after CC. In a group comparison, the small-world organization of patients with good outcomes (groups I and II combined) significantly increased two years after CC, whereas it showed a decreasing trend in patients with bad outcomes. The general functional network pattern changes between pre-operation and post-operation are plotted schematically in Fig. 4D: the high-clustering structures in preoperative states were focused around the midline region and moved to more lateral locations after CC, and the clusters were closely associated with the hubs.

## Discussion

We investigated the long-term functional network effects of CC in patients with LGS and compared the results between patients with different seizure outcomes. First, we calculated surrogate-corrected mutual information (MI) matrices of the preoperative and 3-month, 1-year, and 2-year postoperative EEGs and plotted their network topologies. For quantitative comparisons of network connectivity, the patients were classified into four groups based on preoperative daily seizure frequency and two-year postoperative seizure reduction ratio. Local network structure measures (BC and LCC) and global measures (CPL and GCC) were calculated and statistically tested to examine their long-term changes after CC. Finally, small worldness and general network pattern changes were examined.

The location of hubs (defined as the nodes with highest BC values) changed after CC, which essentially reorganized the network topology. This reorganization remained consistent for the two years after surgery (Fig. 1A and B). The functional connectivity changes were associated with changes in location of the hubs and large-scale clusters (Fig. 4D). This implies that CC could be regarded as a surgical network therapeutic approach, which dissects the preoperative paths to reduce the severity of LGS.

Local network measures were used to evaluate the influence and functional integrity of individual nodes within the network. BC and LCC values across the channels showed little variance over the two-year postoperative follow-up period (Supplementary Figure S1). The lower data dispersion among different cerebral regions reflects a network with more uniform global connectivity in the postoperative state. Because BC is defined as the fraction of shortest paths that pass through a specific region^5^, it was used to evaluate the capacity of bridging nodes connecting disparate parts of the network. Since the dissection of the corpus callosum eliminated many short paths, it is reasonable to assume that the BC would decrease after CC. However, new routes for wide-range communication paths could arise over time, and this could result in increased BC, especially at the two-year follow-up (Fig. 3B group I).

LCC is an indicator of the presence of clusters within a network and quantifies the brain segregation level. As with BC, the reduction in variance over channels in the postoperative state implies more uniform clusters throughout the brain. This could be interpreted as a loss of large-scale clusters around the paramedian region and reorganization toward more homogeneous network. The two-year decrease in the group with good seizure outcomes could be further interpreted as the disappearance of the strong paramedian clusters, leading to more homogeneous and efficient distant network communication. Generally, a regular network has a higher clustering coefficient, whereas a random network possesses more distant connections manifesting as a lower clustering coefficient^2^. Therefore, this result implies a network topology with lower segregation that shows a transition from a regular to more random network.

Global measures, including CPL and GCC, were computed to quantify the long-term effects of CC from a global perspective. GCC showed a decrease in most patient groups (Fig. 4B). Further, decreased CPL in groups II and IV (Fig. 4A) after CC implies enhanced long-distance communication, which signifies that, although the interhemispheric activity spread was largely disrupted by CC, global network communication became more efficient rather than being weakened. A shorter path length is commonly thought to be associated with a better cognitive state^2^. Small-world topology was calculated to evaluate changes in the balance of network segregation and integration. Compared with the reduced small-worldness in groups with bad seizure outcomes (III and IV), the gradual enhancement of small-world organization after CC in groups with good seizure outcomes (I and II) illustrates that a well-recovered brain network shows improved global communication and reduced local clusters. In the current results, changes in global network parameters were not as significant as those of local parameters, as the pre- and post-operation EEGs organized in different patterns, albeit both showed high clustering and short paths (Fig. 4D). Therefore, as a synthetic parameter, small-world organization quantifies the variation in the balance of network segregation and integration. It should, however, only be considered as a global status indicator, not as a detailed description of network structure. Therefore, a detailed network analysis should combine information about both the small-world organization and other aspects of network topology.

As a network-dissection surgery to control generalized refractory seizures, CC can greatly affect brain network topology. Our results show not only immediate network effects at the three-month postoperative follow-up but also further network changes up to two years post-operation. The network topology evolved into a more homogeneous state postoperatively, which was characterized by less synchronization and a consistently reorganized network pattern. We also showed changes in local and global parameters and gradually increased small-world organization in patients with good seizure outcomes after two years. These network effects demonstrate that CC can efficiently alter the brain network topology at both the local and global levels. In terms of the surgical operation and recovery, all these effects should be associated with long-term CC rehabilitation and could serve as indicators of the recovery process.

From the perspective of brain networks, the available surgical treatment options for patients with LGS can be classified as follows: (1) resection of the epileptogenic source nodes, (2) removal of pathological hubs, and (3) dissection of pathological paths. In patients with generalized refractory LGS with no focal imaging findings, it is usually challenging to apply resective surgery, even though some studies have shown successful outcomes^26–29^. CC dissects the interhemispheric fissure, dividing the corpus callosum at the midline, which is equivalent to disconnecting pathological activity-spreading paths, thus palliating the generalized seizure activity. Disconnection of pathological paths can also affect the location of the hubs, as our results showed that the preoperative hubs located around the paramedian regions were shifted more laterally after CC. These results verified CC as a long-term effective network treatment which could serve as an alternative for patients who fail to show focal lesions. The current study is a step toward the clarification of brain’s immediate and long-term network changes in patients with LGS after CC. These consistent network effects offer some valuable indicators and predictors for future surgical treatment and recovery.

However, there are some limitations to the present study. First, the possible effects of age, sex, medication use and CC type were not considered in this study, which means that subtle effects of non-uniformity group may have affected the reliability of the analyses. Second, analysis of other frequency ranges might show additional network effects which can be explored in future studies. Third, the raw data in this study were recorded from 19-channel surface EEG. Although the feasibility and advantages in graph analysis of this technique have been verified by previous studies, on topics such as aging^30^, PTSD^31^, and post-anoxic encephalopathy^32^, the lack of spatial resolution and presence of scalp myogenic artefacts might still affect the specificity of the results. High-density EEG data, with many recording channels, could circumvent these issues. Fourth, the ictal period was not included in the analysis even though doing so could have produced a different network topology. Fifth, although the small-worldness showed significant changes after CC in the good-recovery group, it could not capture the underlying changes in topology. Hence, a more comprehensive interpretation should refer to both global and local parameters. Sixth, we proposed a novel functional effect of CC through the hub-movement analysis. However, whether this ‘hub dissection’ relates to seizure palliation requires more evidence. Moreover, the size limitation of the patient groups with bad seizure outcomes requires some caution in interpretation.

## Methods

### Patient selection and evaluation

We enrolled 30 patients who underwent CC between 2009 and 2012 at Severance Children’s Hospital of Korea. The patients were selected with the following inclusion criteria: (1) preoperative epileptiform discharges showed typical EEG findings of LGS—generalized slow sharp waves and generalized paroxysmal fast activity with a slow and unorganized background; (2) brain MRI findings showed no definite brain lesions; (3) the patient was not a candidate for focal resection based on clinical judgment and interictal EEG recording; (4) patients showed markedly improved condition after surgery. The study was approved by the institutional review board of Yonsei University, College of Medicine, Seoul, Korea, and written informed consent was obtained from all parents of the children prior to participation. In accordance with approved guidelines, patient information including name, seizure type, EEG findings and postsurgical seizure outcome was anonymized prior to our analysis. Table 1 summarizes the baseline patient characteristics, seizure and CC types, preoperative medications, and findings from preoperative and postoperative evaluations. The surgery outcome, ‘good’ or ‘bad’, was assigned on the basis of the seizure reduction rate at two-year follow-up: a ‘good’ surgical outcome was defined as greater than 80% daily seizure reduction. The 30 patients were then divided into four groups: I, high preoperative seizure frequency and good two-year seizure outcome (High-Good), II, low preoperative seizure frequency and good two-year seizure outcome (Low-Good), III, high preoperative seizure frequency and bad two-year seizure outcome (High-Bad), IV, low preoperative seizure frequency and bad two-year seizure outcome (Low-Bad).

**Table 1:** Clinical characteristics of LGS patients.

NO.	Sex/age	Seizure onset age	Main seizure type	CC type	Preoperative medication	Preoperative EEG	3-month postoperative EEG	3-month seizure outcome	1-year postoperative EEG	1-year seizure outcome	2-year postoperative EEG	2-year seizure outcome
1	M/9	7	Atypical absence	Complete	OXC, LEV, TPM	GPFA, GSSW	GPFA, GSSW▼	60%▼	MSWD	90%▼	MSWD	Seizure free
2	M/2	0.8	Spasms	Partial	VGB, LEV, VPA	GPFA, GSSW	Lt LPFA, Lt SWD	60%▼	Lt SWD	90%▼	Lt SWD▼	Seizure free
3	F/8	0.5	GT	Partial	LEV, VPA, TPA	GPFA, GSSW	Lt LPFA	90%▼	Lt LPFA	90%▼	Bilateral LPFA, MSWD	Seizure free
4	F/4.1	2.1	Tonic	Partial	VPA, LMT, CZM	GPFA, GSSW	Rt F SWD	Seizure free	No EFD	Seizure free	No EFD	Seizure free
5	M/4.6	1.1	Atonic-drop attackGTC	Partial	TPM, LMT, LEV	GPFA, GSSW	MSWD	93%▼	No EFD	Seizure free	Lt T SWD	Seizure free
6	M/13.8	3	Head droptonic	Complete	ZNS, CLB, LEV, TPM	GPFA, GSSW	Rt F SWD	Seizure free	MSWD	Seizure free	MSWD	Seizure free
7	F/12	8	Atonic-drop attackabsence	Complete	LEV, VPA, VGB	GPFA, GSSW	No EFD	Seizure free	No EFD	Seizure free	No EFD	Seizure free
8	F/8	2	GTC, head drop	Complete	LMT, ZNS, LEV, VPA	GPFA, GSSW, MSWD	Rt LPFA, Rt F SWD	Seizure free	MSWD	99%▼	Lt SWD	99%▼
9	M/7	0.8	GT	Partial	ZNS, VPA, LEV	GPFA, GSSW	Lt LPFA	Seizure free	Lt SWD	90%▼	MSWD	99%▼
10	M/15.3	0.8	Repetitive tonic, GTC	Complete	VPA, TPM, LEV	GPFA, GSSW	Rt T SWD	Seizure free	Rt T SWD	99%▼	Rt T SWD	99%▼
11	M/10.6	6	Absence, atonic	Partial	CLB, LEV, VPA	GPFA, GSSW	Rt F SWD	Seizure free	Rt F SWD	99%▼	Rt F SWD	98%▼
12	F/18.2	5	Atonic, tonic	Complete	LMT, ZNS	GPFA, GSSW	Lt SWD	Seizure free	Lt SWD	Seizure free	No EFD	98%▼
13	F/7.3	0.3	Atonic-drop attack tonic	Complete	CLB, VGB	GPFA, GSSW	MSWD, Lt LPFA	50%▼	Lt SWD	99%▼	MSWD	96%▼
14	F/15	1.6	GTC, jerking	Partial	TPM, OXC, VGB	GPFA, GSSW	Lt F SWD	95%▼	Lt F SWD	99%▼	Rt F SWD	95%▼
15	M/13	9	Head drop	Partial	TPA, LMT, LEV, VPA	GPFA, GSSW	MSWD	99%▼	MSWD	90%▼	Lt SWD, MSWD	90%▼
16	F/7.8	4	Myoclonictonic	Complete	LMT, LEV, CLB	GPFA, GSSW	Rt F SWD	90%▼	Rt F SWD	98%▼	Rt F SWD	90%▼
17	M/4	0.5	GTC, jerking	Complete	VGB, LEV	GPFA, GSSW	GPFA, GSSW▼	90%▼	Lt SWD	90%▼	GSSW▼	80%▼
18	M/5	0.5	GT, head drop	Complete	LEV, CBZ	GPFA, GSSW	Lt LPFA	90%▼	MSWD	80%▼	GPFA, GSSW▼	80%▼
19	F/1	0.3	Spasms	Partial	TPM, VPA	GPFA, GSSW	GPFA, GSSW▼	50%▼	GPFA, GSSW▼	50%▼	MSWD	80%▼
20	M/10	6	Atypical absence, head drop	Partial	LMT, LEV, VPA	GPFA, GSSW	GPFA, GSSW▼	90%▼	GPFA, GSSW▼	90%▼	Rt SWD	80%▼
21	F/2.4	1	Atonic-drop attacktonic, absence	Partial	ZNS, LEV, VPA	GPFA, GSSW	GPFA, GSSW▼	86%▼	GPFA, GSSW▼	80%▼	Bilateral SWD	76%▼
22	M/12.8	8	Absence, atonic, GT	Partial	LMT, CLB, LEV, TPM	GPFA, GSSW	Rt FT SWD, Rt LPFA	90%▼	Rt FT SWD, Rt LPFA	66%▼	Bilateral SWD	66%▼
23	M/5.9	0.5	atonic, tonic-drop attackmyoclonic	Complete	LEV	GPFA, GSSW	Lt F SWD, GPFA▼	93%▼	Rt LPFA, MSWD	76%▼	GPFA, GSSW▼	66%▼
24	M/12	8	GTC, head drop	Partial	LMT, LEV, TPM	GPFA, GSSW	Rt LPFA	Seizure free	Lt F SWD	50%▼	GSSW▼	50%▼
25	F/4	0.2	GT, SMA seizure	Partial	ZNS	GPFA, GSSW	GPFA, GSSW▼	No change	GPFA, GSSW▼	50%▼	F SWD	50%▼
26	F/4.9	0.2	repetitive tonic	Complete	CLB, ZNS	GPFA, GSSW	bilateral SWD	60%▼	bilateral SWD	20%▼	bilateral SWD	40%▼
27	F/6	0.1	Spasms	Partial	LMT, TPM	GPFA, GSSW	Lt LPFA	30%▼	Lt SWD, MSWD	30%▼	Lt SWD	40%▼
28	F/6.9	0.1	myoclonictonic	Partial	LMT, TPM	GPFA, GSSW	Lt SWD, Rt O SWD	33%▼	Lt SWD, Rt O SWD	66%▼	Lt SWD, Rt O SWD, GSWD▼	33%▼
29	F/2.3	0.1	atonic-drop attackmyoclonic, tonic	Partial	CLB, TPM, LEV	GPFA, GSSW	bilateral SWD	50%▼	bilateral SWD, LPFA, GSWD	0%▼	bilateral SWD	30%▼
30	M/14.8	10	Atonic, tonic-drop attack	Partial	LMT, LEV, VPA	GPFA, GSSW	Lt LPFA, MSDW	99%▼	Lt LPFA, MSDW	50%▼	MSWD	16%▼

F, female; M, male; Lt, left; Rt, Right; F, frontal, T, temporal, O, occipital

GT, generalized tonic; GTC, generalized tonic-clonic;

CBZ, carbamazepine; CLB, clobazam; LEV, Levetiracetam; LMT, lamotrigine; OXC, Oxcarbazepine; RFM, rufinamide; TPM, Topiramate; VGB, Vigabatrin; VPA, Valproate; ZNS, Zonisamide; TPM, Topamax (topiramate); CZM, Rivotril (clonazepam);

GPFA, generalized paroxysmal fast activity; GSSW, generalized slow sharp and wave; LPFA, localized paroxysmal fast activity; MSWD, multifocal sharp and wave discharge; SWD, sharp and wave discharges; ▼, reduction in frequency.

All seizure outcomes are in comparison to the preoperative state.

### EEG acquisition and pre-processing

**Figure 5:**
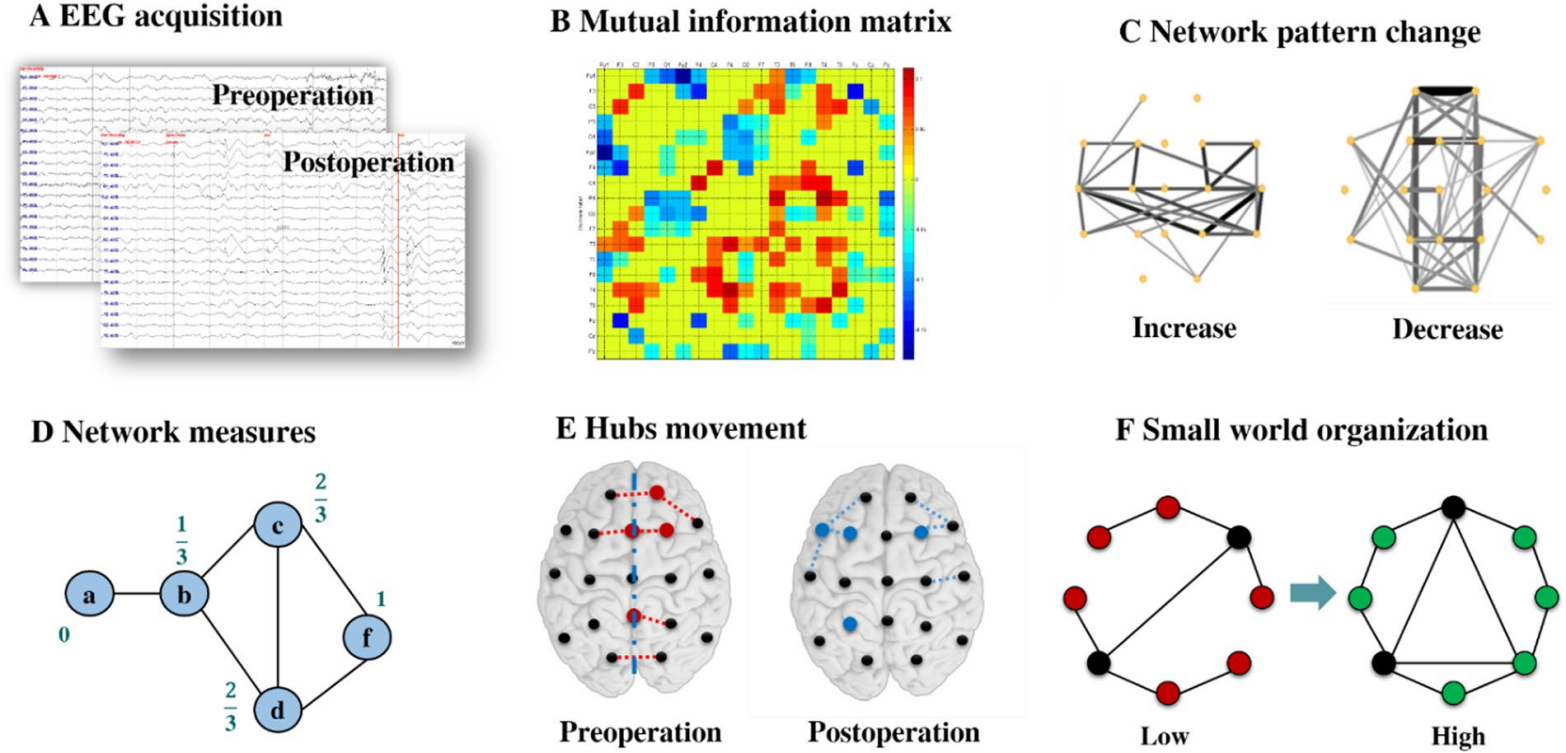
General flow diagram of the network effects analysis.

Patients’ surface-EEG data were recorded with a 19-channel digital EEG acquisition system (Telefactor, Grass Technologies) preoperatively and at three-months, one-year, and two-years post-operation (Fig. 5A). All EEG recordings were performed prior to our analysis as part of the clinical diagnosis and treatment of each patient. The sampling frequency was set to 200 Hz, and the data were re-referenced offline to the average of all channels.

Because the subjects involved in this study all had refractory generalized epilepsy, it was challenging to select a long-term contiguous interictal EEG recording without pathologic waves, especially in the presurgical state. Segmentation into short, one-second epochs could, however, allow artefacts and epileptiform discharges to be excluded while balancing signal stationarity. Generally, a larger raw sampling data volume will form a more accurate and stable network template^33^. Therefore, we randomly selected 300 one-second artefact- and spike-free EEG epochs recorded from resting patients for each testing period. The data were then bandpass filtered from 1–70 Hz and notch filtered at 60 Hz to remove both DC components and line noise, so that the broadband data was selected to minimize type I error while maximizing the amount of usable information^34^. Network topology plots for distinct bands, i.e., delta (1–4 Hz), theta (4–8 Hz), alpha (8–13 Hz), beta (13–32 Hz), and gamma (32–70 Hz), showed connectivity patterns similar to those derived from broadband plots (Supplementary Figure S2), which confirms that broadband analysis could serve as a representative way to study the effects of CC.

### Network construction

Mutual information (MI) has been widely used as a measure of the coupling between two signal streams^35^. We adopted MI as the basis for functional networks because of its ability to characterize short signals with high accuracy and robust estimation despite time delays and non-stationary signals^36,37^. MI was calculated and corrected by surrogate data (described below) for each one-second 19-channel EEG segment, and the adjacency matrix was constructed by averaging 300 epochs for each patient. The matrices were, then, again averaged over each patient group for each testing period (Fig. 5B). We visualized these matrices in connected network graphs using Pajek (http://vlado.fmf.uni-lj.si/pub/networks/pajek/) with various line thicknesses and greyscale representing distinct connection strengths (Fig. 5C). To help better understand the network connection changes, only the upper 30% of the MI values were plotted in this figure. However, network measures were calculated based on the full weighted MI matrix. Broadband networks were analysed separately for preoperative and postoperative EEGs. To characterize the pattern changes from pre-operation to post-operation, the relative changes were expressed by subtracting the matrices for the two recordings.

### Network measures

Several network measures, including local and global measures, were calculated to quantify the centrality of individual regions and the general status of the global network (Fig. 5D). These parameters were calculated based on the weighted MI matrices using the Brain Connectivity Toolbox (http://www.brain-connectivity-toolbox.net/). We then depicted both the preoperative and postoperative hubs visually on a template brain (Fig. 5E). The local measures BC (betweenness coefficient) and LCC (local clustering coefficient) were calculated to quantify the centrality of the local cerebral state. The meaning of each measure is a function of the way it is calculated. BC is defined as the fraction of shortest paths that go through a given node, and therefore quantifies the centrality of a brain region. Thus, a high BC indicates that a region forms a pivotal joint in dynamic communication. LCC measures the level of clustered connectivity around individual nodes, so that a higher LCC value is suggestive of a functional segregation of a certain region in the network^38^. The most important brain regions (hubs) interact with many other regions, facilitate functional integration, and play a central role in network function^38^. In this study, ‘hubs’ were defined as the 5 nodes with the highest BC values.

Global measures, such as characteristic path length (CPL) and global clustering coefficient (GCC), were also calculated for each patient to characterize the global effects of CC. Like the LCC, GCC is a measure that reflects the presence and scale of clusters but evaluates the level of segregation of the global network. CPL calculation is based on the network paths, which are the routes of information flow between pairs of brain regions. The metrics used were normalized with reference to random models built by MATLAB Tools for Network Analysis (http://strategic.mit.edu). The small-worldness was defined for each group as the ratio of GCC to CPL (Fig. 5F)^14,39^.

### Statistical analysis

#### Surrogate test

For testing the statistical significance of MI results, surrogate datasets consisting of 1000 data points for each pair were generated by randomly and independently rearranging the phases of the time series. The empirical distributions of the MI values for the surrogate data were used to determine the *p* = 0.05 significance threshold.

#### Levene’s test

To evaluate the homogeneity of the global brain network connectivity, Levene’s test was used to compare local network parameters at pre-operation and three months post-operation, using the MATLAB function “Levene test” published by Antonio Trujillo-Ortiz (MathWorks MATLAB Central).

#### RM-ANOVA

To test the significance of the computed network measures results over follow-up periods, all local and global measures were subjected to a repeated measures ANOVA for each group (I, II, III, and IV), treating measurement time as a within-subject factor. We subsequently ran post hoc tests with Bonferroni corrections for the comparisons that were found significant. The *p* value 0.05 was used as significance level for all tests.

### Data availability

The datasets generated and analysed during the current study are available from the corresponding author on reasonable request.

## Electronic supplementary material

Supplementary Information
