# Supplementary material for: Changes in functional brain network topology after successful and unsuccessful corpus callosotomy for Lennox-Gastaut Syndrome
Source: Sci Rep. 2018 Feb 21;8:3414. doi: 10.1038/s41598-018-21764-5 (PMC5821858; doi:10.1038/s41598-018-21764-5)
Supplement: Supplementary file 1 — Supplementary Information [file 41598_2018_21764_MOESM1_ESM.doc]

# Changes in functional brain network topology after successful and unsuccessful corpus callosotomy for Lennox-Gastaut Syndrome

Jun-Ge Liang^1^, Nam-Young Kim^1*^, Ara Ko^3^, Heung Dong Kim^2, 3*^，Dongpyo Lee^2*^


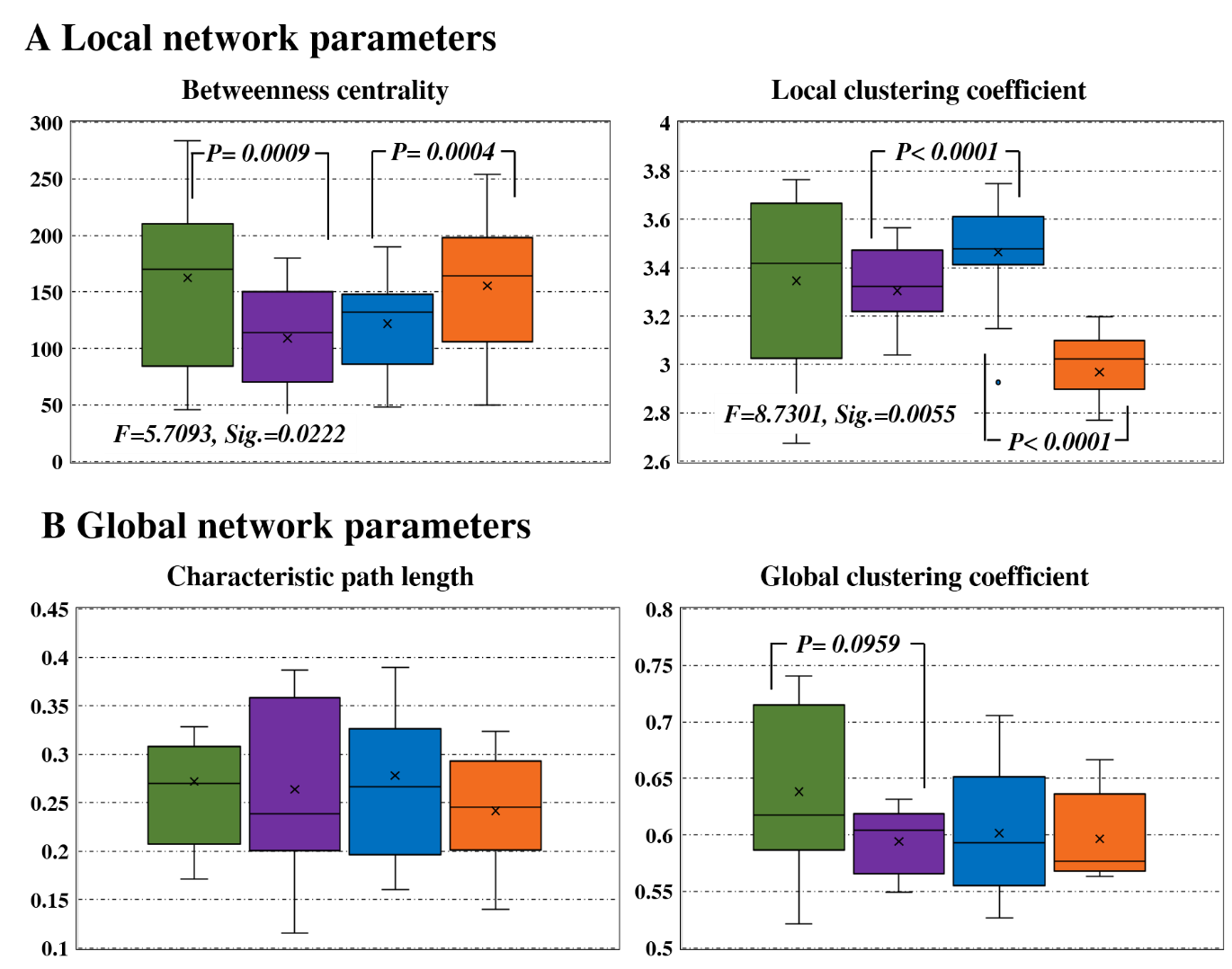


Supplementary Figure S1. Local network parameters including betweenness centrality and local clustering coefficients based on all 30 subjects. The decreased intra-group data variance verified by Levene’s tests for BC (F = 5.7093, Sig.= 0.0222) and LCC (F = 8.7301, Sig.= 0.0055).


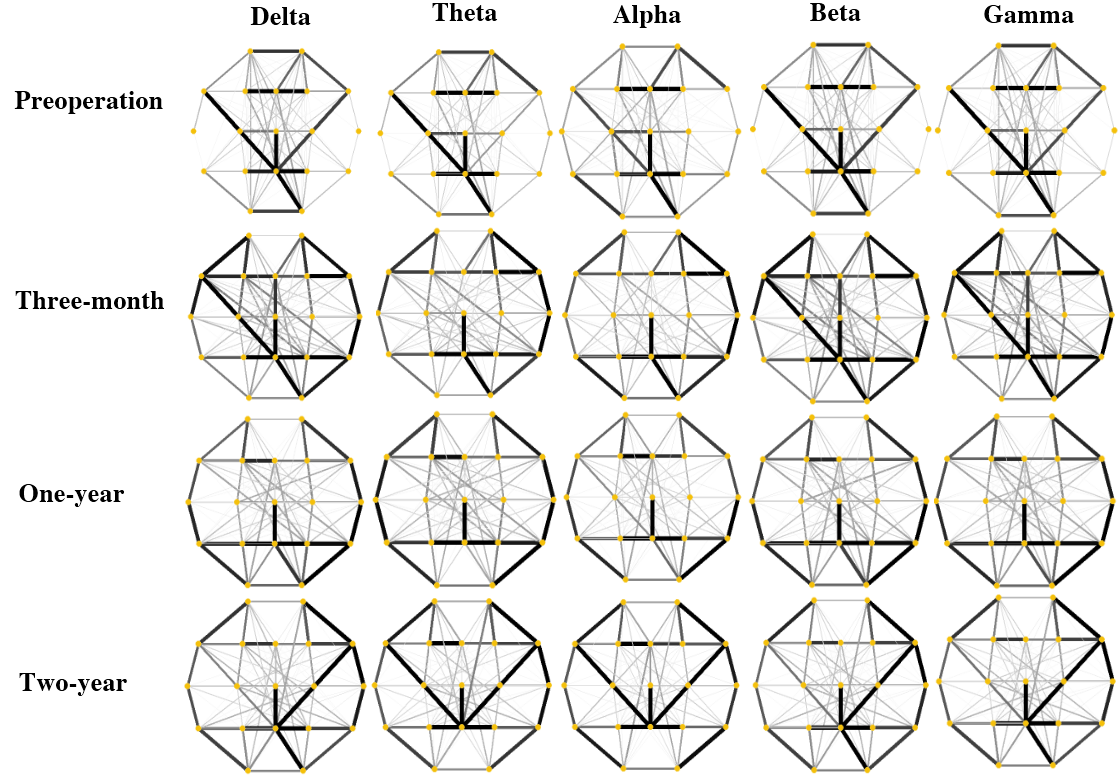


(A)


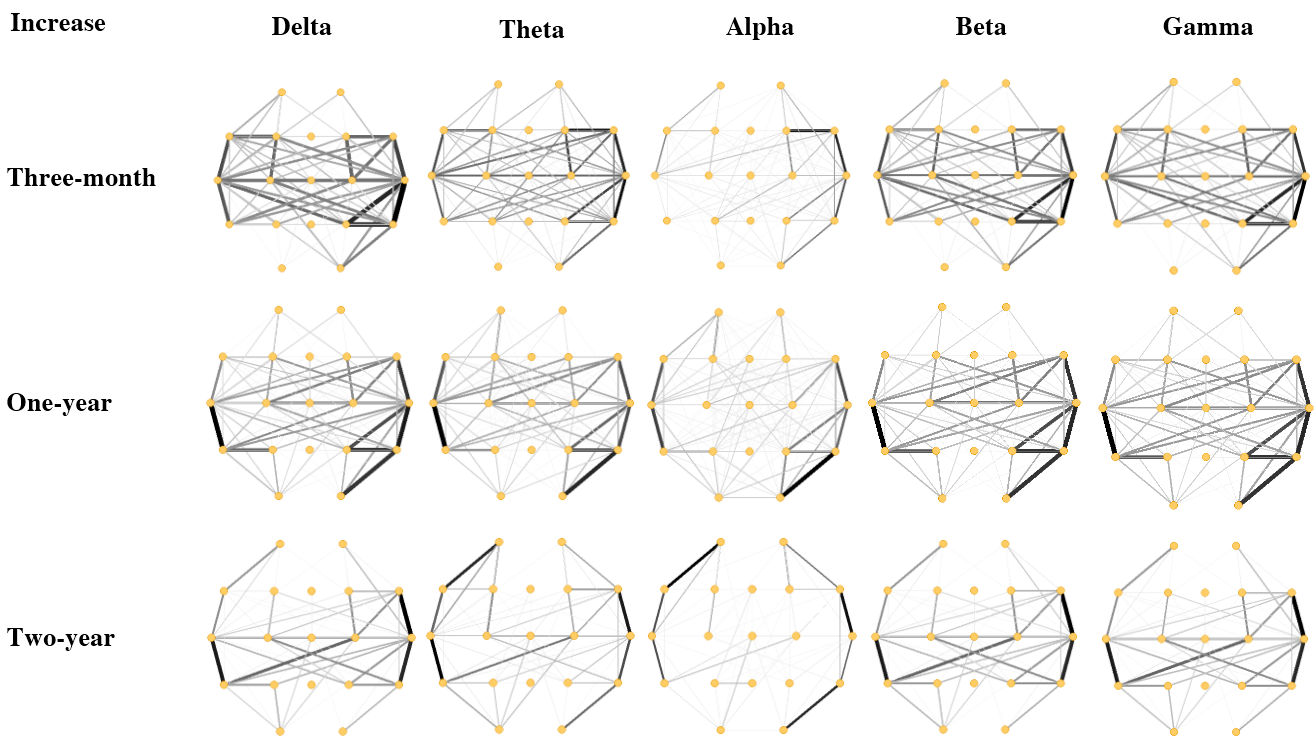


(B)


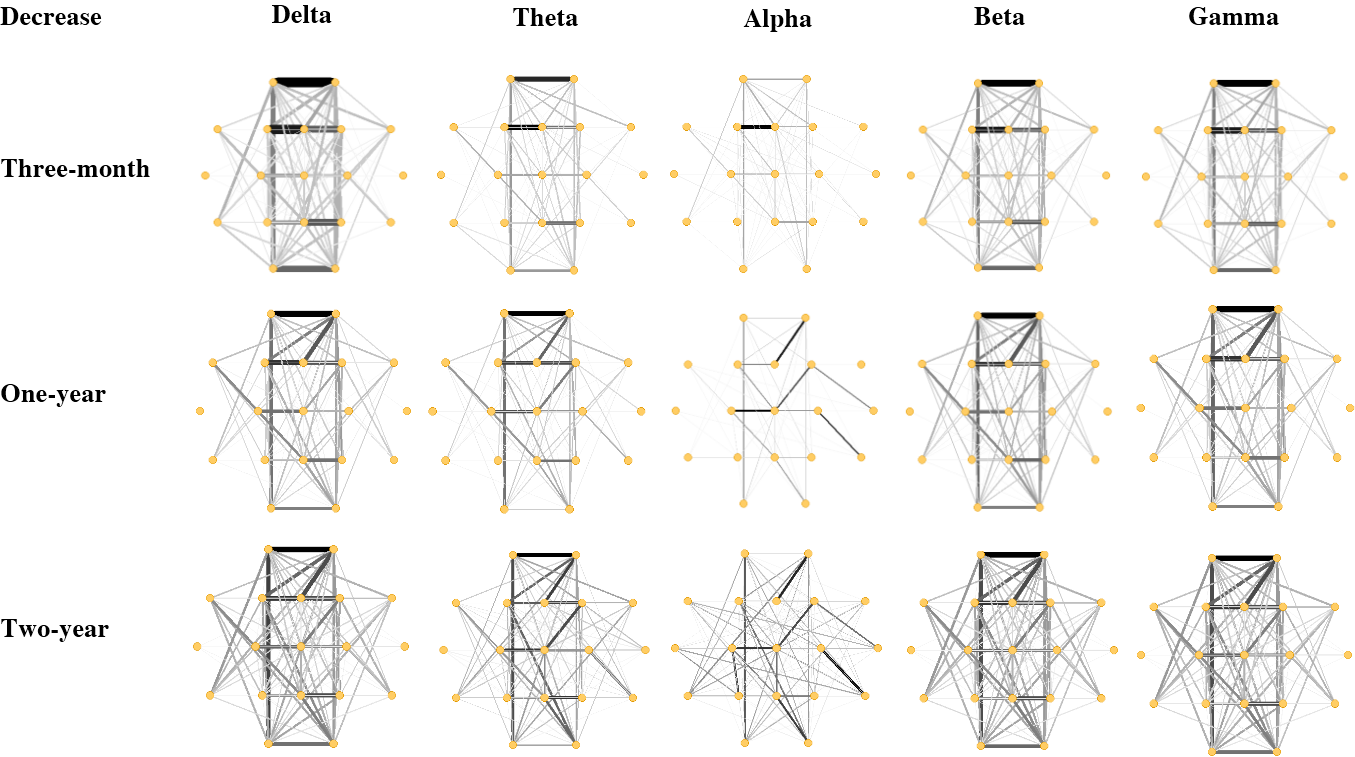


(C)

Supplementary Figure S2. (A) Separated frequency band network topologies in pre-operation and at three-month, one-year, and two-year postoperative states, and (B) increased and (C) decreased network connections at these three states versus pre-operation.


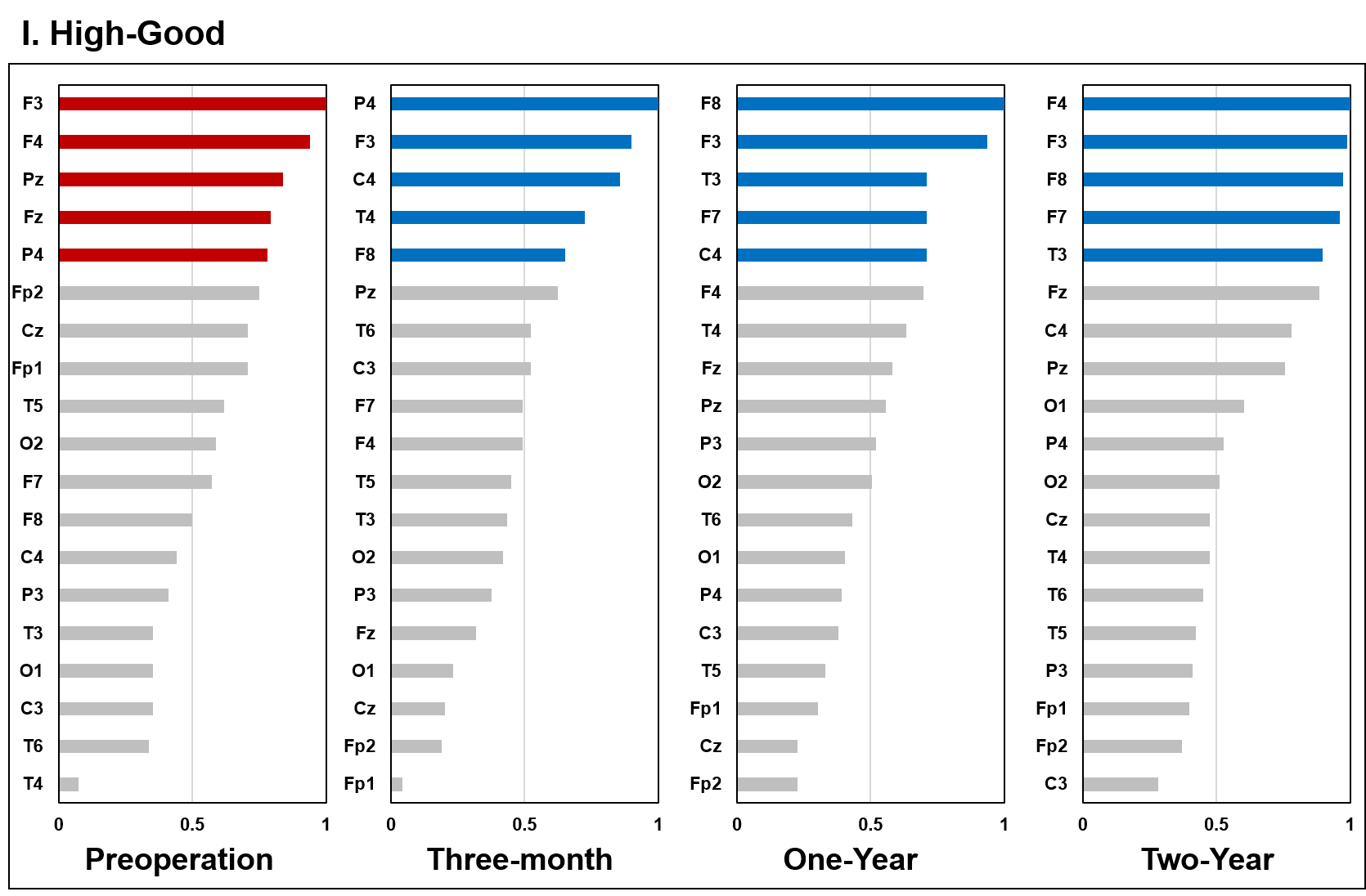


(A)


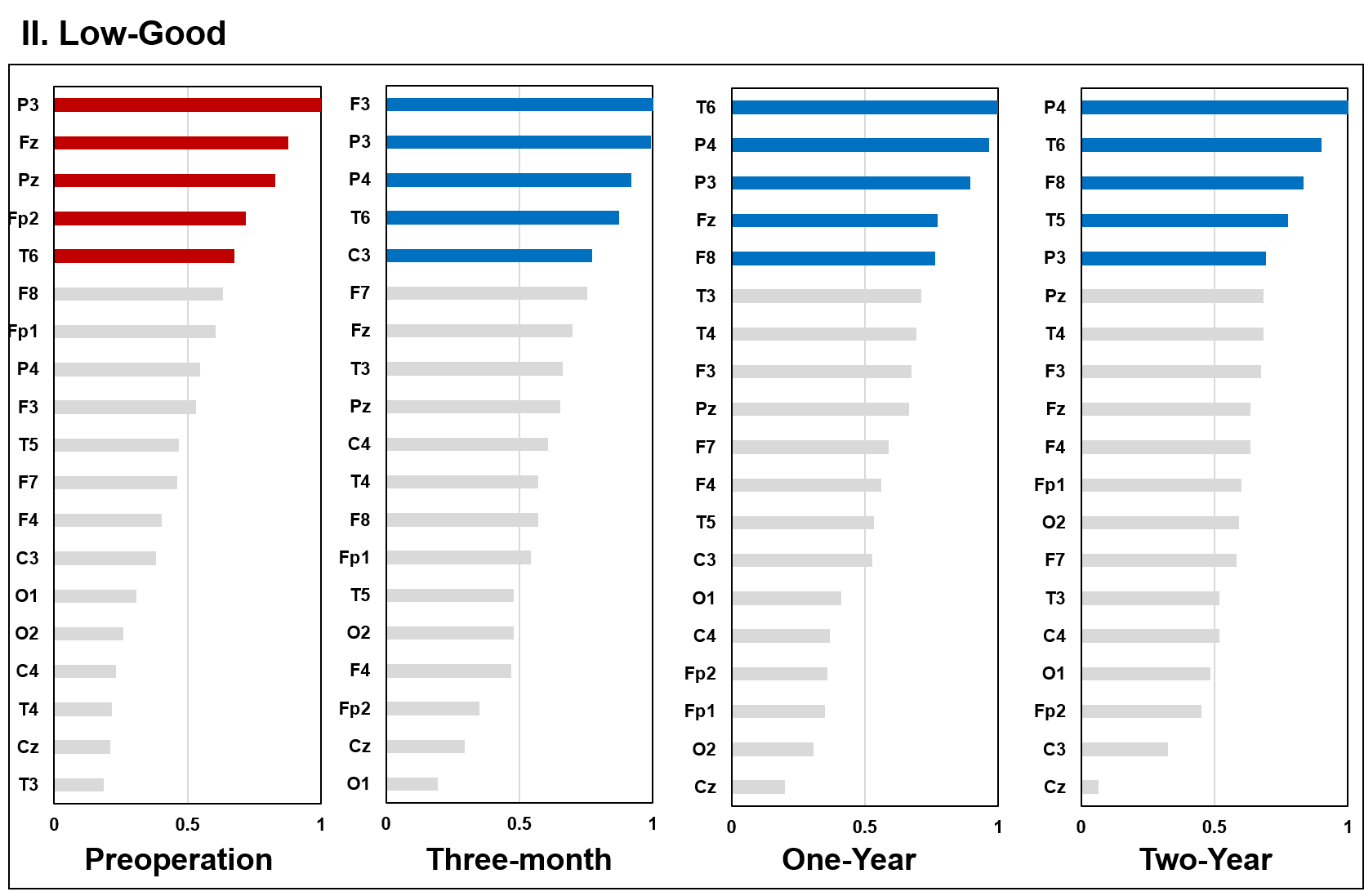


(B)


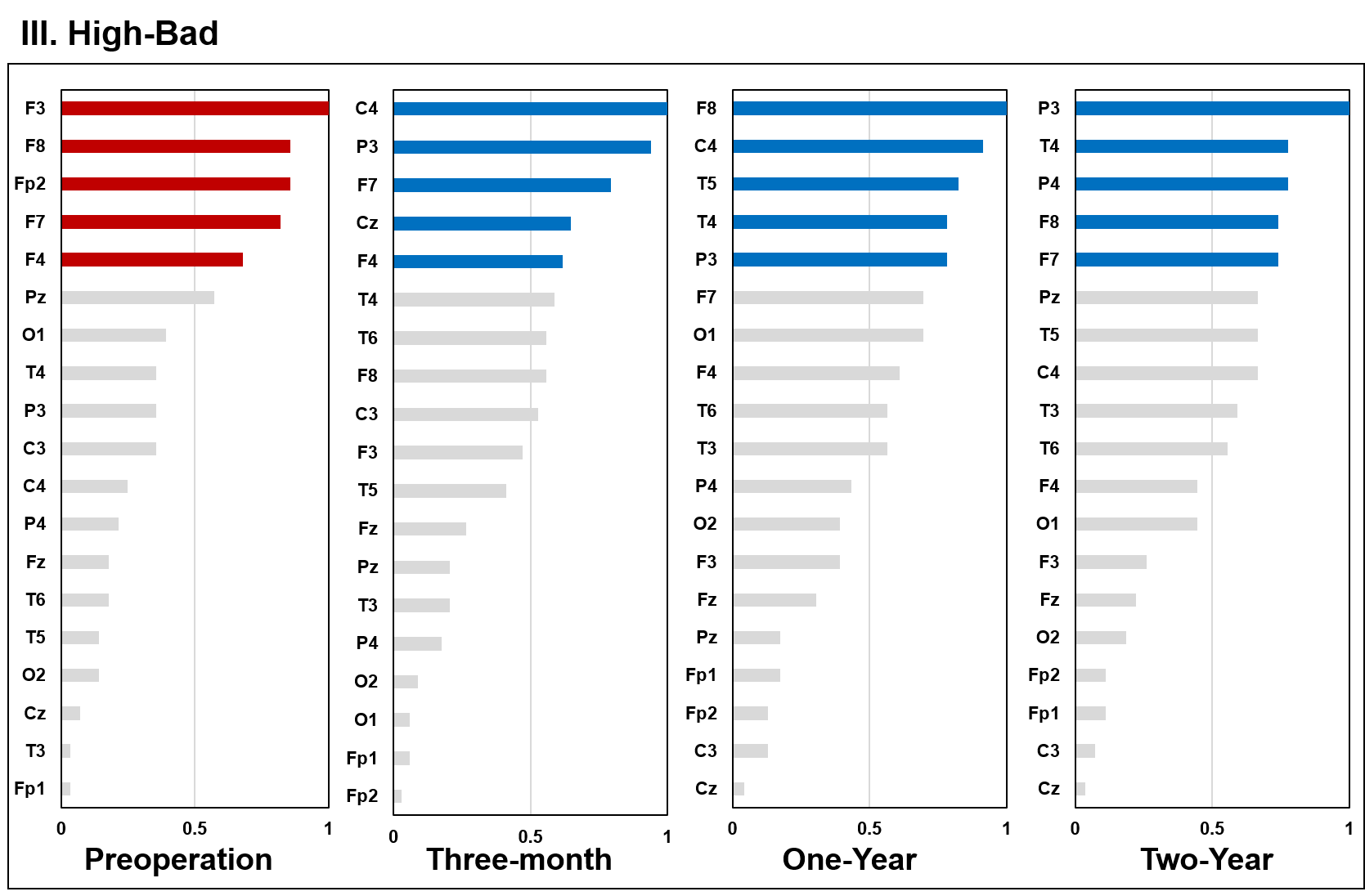


(C)


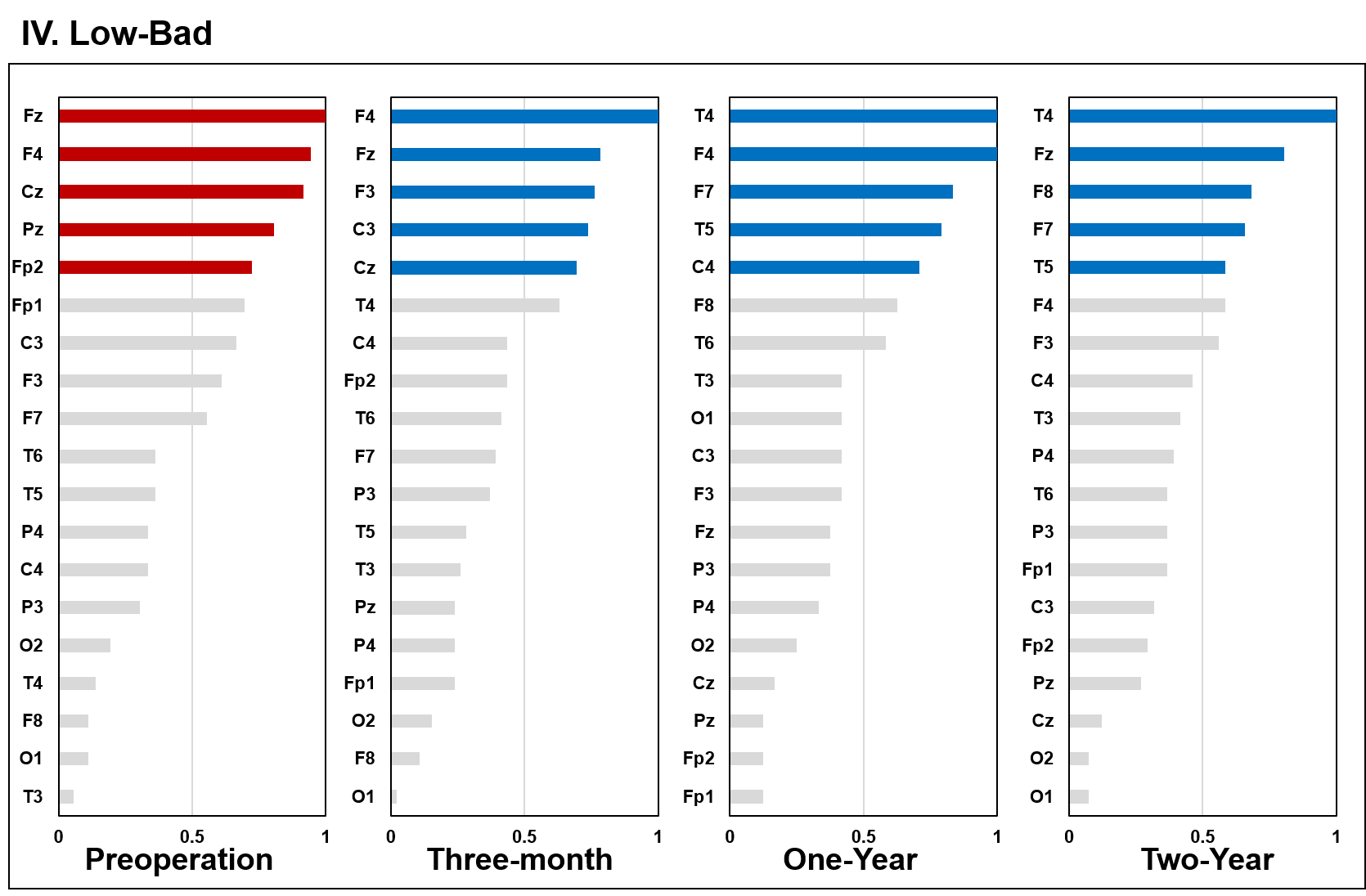


(D)

Supplementary Figure S3. Normalized betweenness centrility of all 19 channels with the top five vules indicating the hubs (red color: preoperative hubs, blue color: postoperative hubs), (A) I. High-Good, (B) II, Low-Good, (C) III, High-Bad, (IV) Low-Bad.
